# Hospitalization and emergency department visits associated with potentially inappropriate medication in older adults: self-controlled case series analysis

**DOI:** 10.3389/fpubh.2023.1080703

**Published:** 2023-06-30

**Authors:** Jaeok Lim, Sohyun Jeong, Suhyun Jang, Sunmee Jang

**Affiliations:** ^1^College of Pharmacy and Gachon Institute of Pharmaceutical Sciences, Gachon University, Incheon, Republic of Korea; ^2^Hinda and Arthur Marcus Institute for Aging Research, Hebrew SeniorLife, Boston, MA, United States; ^3^Department of Medicine, Beth Israel Deaconess Medical Center, Harvard Medical School, Boston, MA, United States

**Keywords:** potentially inappropriate medication, self-controlled case series, Poisson regression, older adult, pain medication, gastrointestinal medication, anticholinergics

## Abstract

**Introduction:**

Potentially inappropriate medications (PIM) and resulting adverse health outcomes in older adults are a common occurrence. However, PIM prescriptions are still frequent for vulnerable older adults. Here, we sought to estimate the risk of hospitalization and emergency department (ED) visits associated with PIM prescriptions over different exposure periods and PIM drug categories.

**Methods:**

We used the National Health Insurance Service-Elderly Cohort Database (NHIS-ECDB) to construct the cohort and implemented a Self-Controlled Case Series (SCCS) method. Hospitalization or ED visits during the exposure and post-exposure periods were compared to those during the non-exposure period, and six PIM drug categories were evaluated. A conditional Poisson regression model was applied, and the risk of outcomes was presented as the incidence rate ratio (IRR). All potential time-varying covariates were adjusted by year. A total of 43,942 older adults aged ≥65 y who had at least one PIM prescription and the events of either hospitalization or ED visits between Jan 2016 and Dec 2019 were selected..

**Results:**

Mean days of each exposure period was 46 d (±123); risk was highest in exposure1 (1–7 d, 37.8%), whereas it was similar during exposure2 (15–28 d), and exposure3 (29–56 d) (16.6%). The mean number of total PIM drugs administered during the study period was 7.34 (±4.60). Both hospitalization and ED visits were significantly higher in both exposure (adjusted IRR 2.14, 95% Confidence Interval (CI):2.11–2.17) and post-exposure periods (adjusted IRR 1.41, 95% CI:1.38–1.44) in comparison to non-exposure period. The risk of adverse health outcomes was highest during the first exposure period (1–14 d), but decreased gradually over time. Among the PIM categories, pain medication was used the most, followed by anticholinergics. All PIM categories significantly increased the risk of hospitalization and ED visits, ranging from 1.18 (other PIM) to 2.85 (pain medication). Sensitivity analyses using the first incidence of PIM exposure demonstrated similar results. All PIM categories significantly increased the risk of hospitalization and ED visits, with the initial period of PIM prescriptions showing the highest risk. In subgroup analysis stratified by the number of medications, PIM effects on the risk of hospitalization and ED visits remained significant but gradually attenuated by the increased number of medications.

**Discussion:**

Therefore, the development of deprescribing strategies to control PIM and polypharmacy collectively is urgent and essential.

## 1. Introduction

Worldwide, the proportion of adults aged ≥60 y is increasing dramatically. One in six people in the world will be aged 60 y or older by 2030, and their population will double by 2050 (2.1 billion) (1). Older adults have an increased risk of adverse drug reactions due to age-dependent changes in pharmacokinetics and pharmacodynamics as well as polypharmacy and complex drug regimens based on increased susceptibility to chronic complex diseases (2). As a result, drug-related problems are an important health care safety concern for older people. Potentially inappropriate medications (PIMs) are defined as those with a greater risk of harm than benefit, particularly in patients older than 65 y (3). The prevalence of PIMs in older adults ranges from 20 to 60% based on the healthcare settings or criteria used to define PIM (Beers Criteria^®^ or STOPP criteria) (4). Potentially inappropriate medication prescriptions are associated with 10 to 30% increased risk of hospitalization (5–9), increased risk of adverse drug events (ADEs) (10–12), emergency department (ED) visits (13, 14), and a poor health status (15). In addition, PIMs directly or indirectly increase healthcare use and costs (16). However, a Japanese study using the 2012 Beers criteria found no relationship between PIM exposure and adverse outcomes (17). Adverse health outcomes associated with PIM use should be associated with the number and types of comorbidities in older adults (18, 19), which is usually accounted for as a covariate adjustment, but not taken into full consideration in most studies.

In Korea, over 80% of older adults have experience of PIM consumption, defined by 2012 Beers criteria, according to a 2009–2011 study using Health Insurance Review and Assessment (HIRA) database (20) and their use is recurrent and consistent in many cases. Therefore, finding a control group and assessing the precise outcomes accordingly is difficult, as determining prescription days is complicated.

In this context, we implemented a Self-Controlled Case Series (SCCS) model to fit the characteristics of PIM use in older adults in Korea and fully implement the meticulous analysis method.

The SCCS method provides an alternative epidemiological study design for investigating the association between transient exposure and outcome events. The SCCS method is a case-only method; it has the advantages that no separate controls are required and any time-invariant confounders, such as comorbidity, are automatically controlled. It also requires precise timings; therefore, the SCCS method is best suited to acute events and transient exposures for which periods of exposure risk can be clearly defined (21).

This study had two main aims: (1) to estimate the risk of adverse health outcomes due to PIM use: hospitalization and ED visits, as well as risk stratification based on different risk (exposure) periods (prescription days), and (2) PIM categorization according to the differential risk toward hospitalization and ED visits.

## 2. Materials and methods

### 2.1. Data sources

We used the National Health Insurance Service-Elderly Cohort Database (NHIS-ECDB), a sample research database providing insurance claim information on individuals over the age of 60, starting in 2012, which currently comprises approximately 1,000,000 cases. The NHIS-ECDB provides multiple variables regarding basic demographic information, disability, death, social and economic status, medical service utilization, and long-term nursing home services; details of medical and dental treatment; and prescription information (National Health Insurance Sharing Service, Sample Research DB, https://nhiss.nhis.or.kr/bd/ab/bdaba022Oeng.do#). This study protocol was exempt from review by the Institutional Review Board of Gachon University (IRB number: 1044396-202,005-HR-100-01).

### 2.2. Study design and outcomes

We implemented a SCCS method where study participants act as their own controls, therefore this study included participants who had the exposure (PIM use), non-exposure (PIM non-use) periods and outcome (hospitalization/ED visits) events from Jan 2016 to Dec2019. All exposures occurring within the observation period, both before and after individuals have experienced the event, are included in the analysis. The outcomes of this study were to assess the overall risk of hospitalization or ED visits associated with PIM use stratified by exposure period and the differential risk of each PIM drug category with regard to outcome events.

#### 2.2.1. Study population

The inclusion criteria were as follows: (1) age ≥ 65 y who experienced outcomes of interest, and had at least one prescription of PIM during the study period (Jan 2016–Dec to 2019), (2) no PIM prescriptions 6 months before cohort enrollment (Jul 2015–Dec 2015), and (3) no cancer history during the entire study period. To account for new user qualifications, we defined older adults who had no PIM prescription for 6 months (wash-out period) before cohort enrollment as new PIM users. This criterion was to make sure the outcome events were from incidental PIM use not from carry-over effects from the previous PIM use. Due to the complex disease characteristics and complicated treatment regimens, we excluded older adults who had any cancer history during the study period (removed individuals with ICD-10 codes C00-C97).

#### 2.2.2. Outcome events, exposure, and non-exposure periods

PIM was defined based on the 2019 updated Beers criteria (22). The Beers Criteria are one of the most widely used explicit lists of PIMs for the older adults, originally developed by Beers and colleagues in 1991 through an evidence-based comprehensive literature review and expert panel consensus using the Delphi method (23). We only used PIM categories in Beers criteria 2019 as exposures (Supplementary Table S1), due to the fact that others in Beers criteria 2019 are regarding older adults with specific conditions and specific PIM properties, and aimed to represent the more general PIM use outcomes in older adults. The PIM drugs and categories used in this study, are presented in Supplementary Table S1 (22). The outcomes of interest were combined events of hospitalization and ED visits, and hospitalization and ED visit alone.

**Figure 1:**
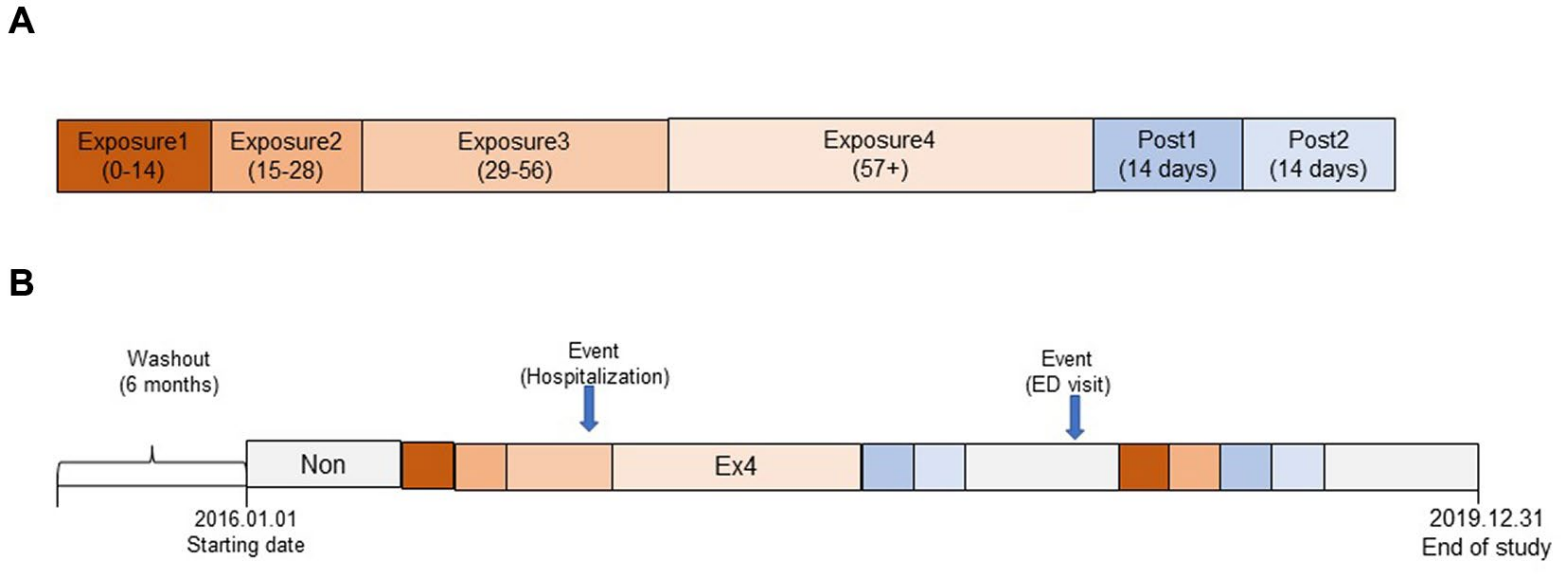
Overall conceptual model of the self-controlled case study design (SCCS) in this study. **(A)** Segmentation of exposure and post-exposure period. **(B)** Schematic representation of observation period (example).

We stratified the exposure periods into exposure1 (day_0_ ~ day_14_), 2 (day_15_ ~ day_28_), 3 (day_29_ ~ day_56_), and 4 (day_57_ ~ day_end_), considering the duration of PIM prescription after its initiation. We also defined the post-exposure period as post1 (day_end+1_–day_end+14_) and post2 (day_end+15_–day_end+28_) to account for the residual effects of the PIM. The period when there was no PIM prescription, before or after the exposure, was defined as the non-exposure period after excluding exposure and post-exposure periods. The follow-up period continued until December 2019, but older adults who died before the study end date were observed until the death event. The overall study design is depicted in Figure 1.

### 2.3. Statistical analysis and covariates definition

The baseline characteristics of the included older adults were represented by descriptive statistics by numbers and percentages in discrete variables or mean and standard deviation in continuous variables (Table 1). In our SCCS study design, study participants acted as their own controls, thus all time-invariant covariates within individuals were controlled. We estimated the risk of hospitalization and ED visits by comparing the incidence rates of the outcome between the exposure/post-exposure and non-exposure periods within the same individuals using a conditional Poisson regression model and presented the crude incidence rate ratio (IRR). The IRR was calculated by dividing number of events by the sum of person-years multiplied in each period, and the 95% confidence intervals (CIs) were calculated using a Poisson distribution. Since the study period was 3 y, we used time-variant covariate information that could potentially affect the outcome events by year into adjusted models, such as age group, insurance type, long-term care eligibility, disability, Charlson Comorbidity Index (CCI), comorbidity, and comedication (antithrombotics and systemic steroids), and presented the adjusted IRR (aIRR). Comorbidity information was retrieved and confirmed with a previous 1 y history of ICD-10 codes (Supplementary Table S2) or two or more prescriptions specific to those comorbidities. For psychiatric diseases, which are assumed to be highly confidential in Korea, the data were not available to the researchers, so we used medication history to detect psychiatric diseases as comorbidities. For co-medications, we used the previous 1-month history as a definition period, which is reasonably assumed period to affect the outcomes of interest. The number of co-medications was also assessed 1 month before cohort enrollment. After excluding all PIM drugs used in the study as exposures, the remaining antithrombotics and systemic corticosteroids were selected as potential medications that might affect outcomes. All analyses were performed using SAS version 9.4 and significance was determined with two-sided 95% CI and a *p* value <0.05.

**Table 1:** Baseline characteristics of study population (*N* = 43,942).

Variable	Category	
Sex (*n*, %)	Female	22,692 (51.64)
	Male	21,250 (48.36)
Age (mean, sd)		73.71 (6.36)
Age group (*n*, %)	65–74	25,728 (58.55)
	75–84	15,210 (34.61)
	85-	3,004 (6.84)
Insurance (*n*, %)	National health insurance	42,103 (95.38)
	Medical care	2,038 (4.62)
Insurance premium (*n*, %)	0 (no income)	2,653 (6.04)
	1–4	11,204 (25.5)
	5–8	14,592 (33.21)
	9–10 (highest)	15,493 (35.26)
Long-term care insurance beneficiary (*n*, %)		3,566 (8.12)
Disability (*n*, %)		6,478 (14.74)
Death		3,910 (8.9)
CCI score (*n*, %)	0	32,132 (73.12)
	1	8,908 (20.27)
	2+	2,902 (6.6)
Comorbidities (*n*, %)	Hypertension	13,951 (31.75)
	Ischemic heart disease	2,106 (4.79)
	Heart failure	531 (1.21)
	Cerebrovascular disease	2,915 (6.63)
	Diabetes mellitus	3,612 (8.22)
	Chronic kidney disease	562 (1.28)
	Chronic Obstructive Pulmonary Disease	1,969 (4.48)
	Parkinson’s Disease	394 (0.9)
	Arthritis (Rheumatoid and osteoarthritis)	4,180 (9.51)
	Fracture	749 (1.7)
	Hyperlipidemia	10,945 (24.91)
	Dementia	2,221 (5.05)
	Depression	1,182 (2.69)
	Other psychiatric diseases^*^	3,450 (7.85)
Mean number of comedication^**^ (mean, sd)		3.15 (3.60)
Number of comedication (*n*, %)	0	12,622 (28.72)
	1–4	19,136 (43.55)
	5–9	10,222 (23.26)
	10+	1,962 (4.44)
Comedication (*n*, %)	Antithrombotics (coagulation, platelet)	9,880 (22.5)
	Systemic steroid	1,130 (2.57)

*Psychiatric diseases other than depression. **Number of co-medications was assessed 1 month before cohort entry. §Co-medication was assessed 1 month before cohort entry, and antithrombotics and systemic steroids were remaining medications that potentially affected outcomes after excluding all exposures (PIM drugs).

#### 2.3.1. Sensitivity analysis

Given that older adults in Korea tend to have PIM recurrently and consistently, we performed a sensitivity analysis to assess the robustness of the main outcome of this study by comparing the association of first incidental use of PIM (exposure) and outcome events with the association of overall PIM use, including recurrent or consistent prescriptions and outcome events.

## 3. Results

### 3.1. Baseline characteristics

**Figure 2:**
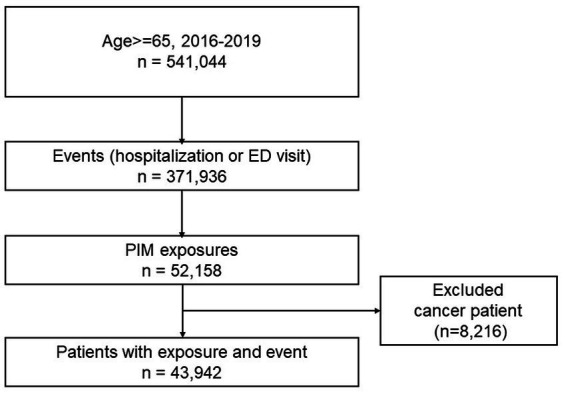
Study population selection flow.

Of the total older adults aged 65 y and older from 2016 to 2019 (*n* = 541,044), 43,942 individuals with PIM exposure and outcomes of hospitalization or ED visits were selected (Figure 2). The number of females was slightly higher (51.62%) than males (48.36%), and 64–74 age group was dominant (58.55%), with a mean age of 73.71 (±6.36). Income status based on medical care (4.62%, categorized as low income) and insurance premium (0–4: no to low income, 31.5%) was observed to be within average. The CCI score showed that the average health status was in good condition (score 0, 73.12%). Long-term care insurance eligible (frail) older adults, and the proportion of disability and death events comprised of 8.12, 14.74, and 8.9%, of individuals, respectively. The predominant comorbidities were hypertension (31.75%), hyperlipidemia (24.91%), and arthritis (both rheumatoid arthritis and osteoarthritis, 9.51%). The proportion of older adults who had polypharmacy (number of co-medications ≥5) was 27.0% (Table 1).

### 3.2. Outcome: overall exposure and outcome events and risk estimation

The mean total number of observation days per individual was 1,352 d, and that of non-exposure days per individual was three times higher than that of the exposure days per individual (940 vs. 260 d). Mean days of each exposure period was 46 d (±123); the highest risk was observed on days 1–7 (37.8%), the risk was similar on 8–14 d and 15–30 d at 16.6%, while the lowest risk was observed at 60 d and over (13.9%). The mean number of PIM drugs prescribed over the whole study period, regardless of their categories per individual was 7.34 (±4.60). Number of PIM drugs in each exposure period was 2.15 (±1.43), and one PIM drug was predominantly prescribed (52.67%) (Table 2).

**Table 2:** Information of exposures per individual older adult (*N* = 43,942).

Variables	
Total observation days (mean, sd)	1,351.6 (247.3)
Total non-exposure days (mean, sd)	940.1 (343.9)
Total exposure days (mean, sd)	260.9 (303.0)
Total post-exposure days (mean, sd)	150.7 (99.3)
Days of each non-exposure period (mean, sd)	168.6 (232.0)
Days of each exposure period (mean, sd)	46.0 (123.3)
Days of each exposure period (*N*, %)	
1–7	94,115 (37.8)
8–14	41,422 (16.6)
15–28	35,479 (14.2)
29–56	37,659 (15.1)
57+	40,509 (16.3)
Days of each post-exposure period (mean, sd)	27.6 (2.7)
Number of exposure periods in total study period per individual (mean, sd)	5.67 (3.59)
Number of PIM drugs in total study period, mean (sd)	7.34 (4.60)
1–4 (*N*, %)	13,792 (31.39)
5–9 (*N*, %)	17,679 (40.23)
10–41 (*N*, %)	12,471 (28.37)
Number of PIM drugs per each exposure period, mean (sd)	2.15 (1.43)
1 (*N*, %)	23,146 (52.67)
2 (*N*, %)	5,094 (11.49)
3–4 (*N*, %)	33,171 (13.30)
5–28	1,659 (3.68)

The mean number of outcomes per individual was as follows: hospitalization, ED visits, and both were 1.87(±1.78), 0.48 (±1.86), and 2.34 (±2.54), respectively. A total of 3,910 (8.90%) death events occurred during the study period, and mean days to death was 867.5 (±379.6) d. In the Poisson regression analysis, both hospitalization and ED visits were significantly higher during the exposure period (IRR 1.99, 95% CI:2.11–2.17), and the post-exposure period (IRR 1.41, 95% CI:1.38–1.44) than the non-exposure period. The risk of ED visits related to PIM use was lower during the post-exposure period than the non-exposure period. All adjusted models using time-variant covariates for each year (age, insurance, income, long-term care insurance eligibility, CCI score, comorbidity, and number of co-medications) presented results similar to those of the unadjusted model (Table 3). We also determined the risk of hospitalization and ED visits according to segmented PIM exposure periods. The risk was the highest during the first exposure period (1–14 d), and decreased over time, including over the post-exposure period (Table 4).

**Table 3:** Risk of hospitalization and ED visit associated with PIM use.

Period	Number of events	Person-years	Incident rate (95% CI)	Crude IRR	Adjusted IRR
All events (*n* = 43,942)					
Non-exposure	56,572	113,098	0.5 (0.5, 0.5)	1	1
Exposure	33,644	31,384	1.07 (1.06, 1.08)	2.14 (2.11, 2.17)	1.99 (1.95, 2.03)
Post-exposure	12,797	18,129	0.71 (0.69, 0.72)	1.41 (1.38, 1.44)	1.41 (1.37, 1.44)
Hospitalizations (*n* = 38,701)					
Non-exposure	44,071	98,711	0.45 (0.44, 0.45)	1	1
Exposure	27,247	28,462	0.96 (0.95, 0.97)	2.14 (2.11, 2.18)	2.01 (1.97, 2.04)
Post-exposure	10,822	15,869	0.68 (0.67, 0.69)	1.53 (1.5, 1.56)	1.55 (1.52, 1.59)
ED visits (*n* = 12,695)					
Non-exposure	12,501	32,661	0.38 (0.38, 0.39)	1	1
Exposure	6,397	9,416	0.68 (0.66, 0.7)	1.77 (1.72, 1.83)	1.69 (1.6, 1.79)
Post-exposure	1,975	5,501	0.36 (0.34, 0.37)	0.94 (0.89, 0.98)	0.89 (0.83, 0.95)

*IRR and 95% CI were estimated using Poisson regression model. §Adjusted IRR was presented by adjusting for age group, insurance, income, long-term care, disability, CCI score, comorbidity, and co-medication information by the year change.

**Table 4:** Risk of hospitalization and ED visits associated with PIM use by segmented exposure period.

Period	Number of events	Person-years	Incident rate (95% CI)	Crude IRR	Adjusted IRR
All events (*n* = 43,942)					
Non-exposure	56,572	113,098	0.5 (0.5, 0.5)	1	1
Exposure1	11,220	6,860	1.64 (1.61, 1.67)	3.27 (3.2, 3.34)	3.29 (3.21, 3.37)
Exposure2	3,855	3,461	1.11 (1.08, 1.15)	2.23 (2.16, 2.3)	2.2 (2.12, 2.28)
Exposure3	4,298	4,048	1.06 (1.03, 1.09)	2.12 (2.06, 2.19)	2.04 (1.97, 2.11)
Exposure4	14,271	17,015	0.84 (0.83, 0.85)	1.68 (1.65, 1.71)	1.46 (1.42, 1.5)
Post1	7,591	9,121	0.83 (0.81, 0.85)	1.66 (1.62, 1.7)	1.69 (1.65, 1.74)
Post2	5,206	9,008	0.58 (0.56, 0.59)	1.16 (1.12, 1.19)	1.17 (1.14, 1.21)
Hospitalizations (*n* = 38,701)					
Non-exposure	44,071	98,711	0.45 (0.44, 0.45)	1	1
Exposure1	9,110	6,053	1.5 (1.47, 1.54)	3.37 (3.3, 3.45)	3.39 (3.31, 3.47)
Exposure2	3,104	3,089	1.01 (0.97, 1.04)	2.25 (2.17, 2.33)	2.22 (2.14, 2.31)
Exposure3	3,481	3,652	0.95 (0.92, 0.98)	2.13 (2.06, 2.21)	2.06 (1.99, 2.14)
Exposure4	11,552	15,668	0.74 (0.72, 0.75)	1.65 (1.62, 1.69)	1.45 (1.41, 1.49)
Post1	6,460	7,984	0.81 (0.79, 0.83)	1.81 (1.77, 1.86)	1.85 (1.79, 1.9)
Post2	4,362	7,885	0.55 (0.54, 0.57)	1.24 (1.2, 1.28)	1.26 (1.22, 1.3)
ED visits (*n* = 12,695)					
Non-exposure	12,501	32,661	0.38 (0.38, 0.39)	1	1
Exposure1	2,110	2,066	1.02 (0.98, 1.06)	2.67 (2.55, 2.79)	2.65 (2.48, 2.82)
Exposure2	751	1,031	0.73 (0.68, 0.78)	1.9 (1.77, 2.05)	1.89 (1.72, 2.07)
Exposure3	817	1,200	0.68 (0.63, 0.73)	1.78 (1.66, 1.91)	1.74 (1.59, 1.9)
Exposure4	2,719	5,119	0.53 (0.51, 0.55)	1.39 (1.33, 1.45)	1.27 (1.19, 1.37)
Post1	1,131	2,768	0.41 (0.38, 0.43)	1.07 (1, 1.13)	1.06 (0.98, 1.14)
Post2	844	2,733	0.31 (0.29, 0.33)	0.81 (0.75, 0.87)	0.79 (0.73, 0.87)

*IRR and 95% CI were estimated using Poisson regression model. §Adjusted IRR was presented by adjusting for age group, insurance, income, long-term care, disability, CCI score, comorbidity, and co-medication information by the year change. #Exposure1(day0–day14), exposure2 (day15–day28), exposure3 (day29–day56), and exposure4 (day57–day_end).

### 3.3. Risk estimation by PIM drug categories

**Figure 3:**
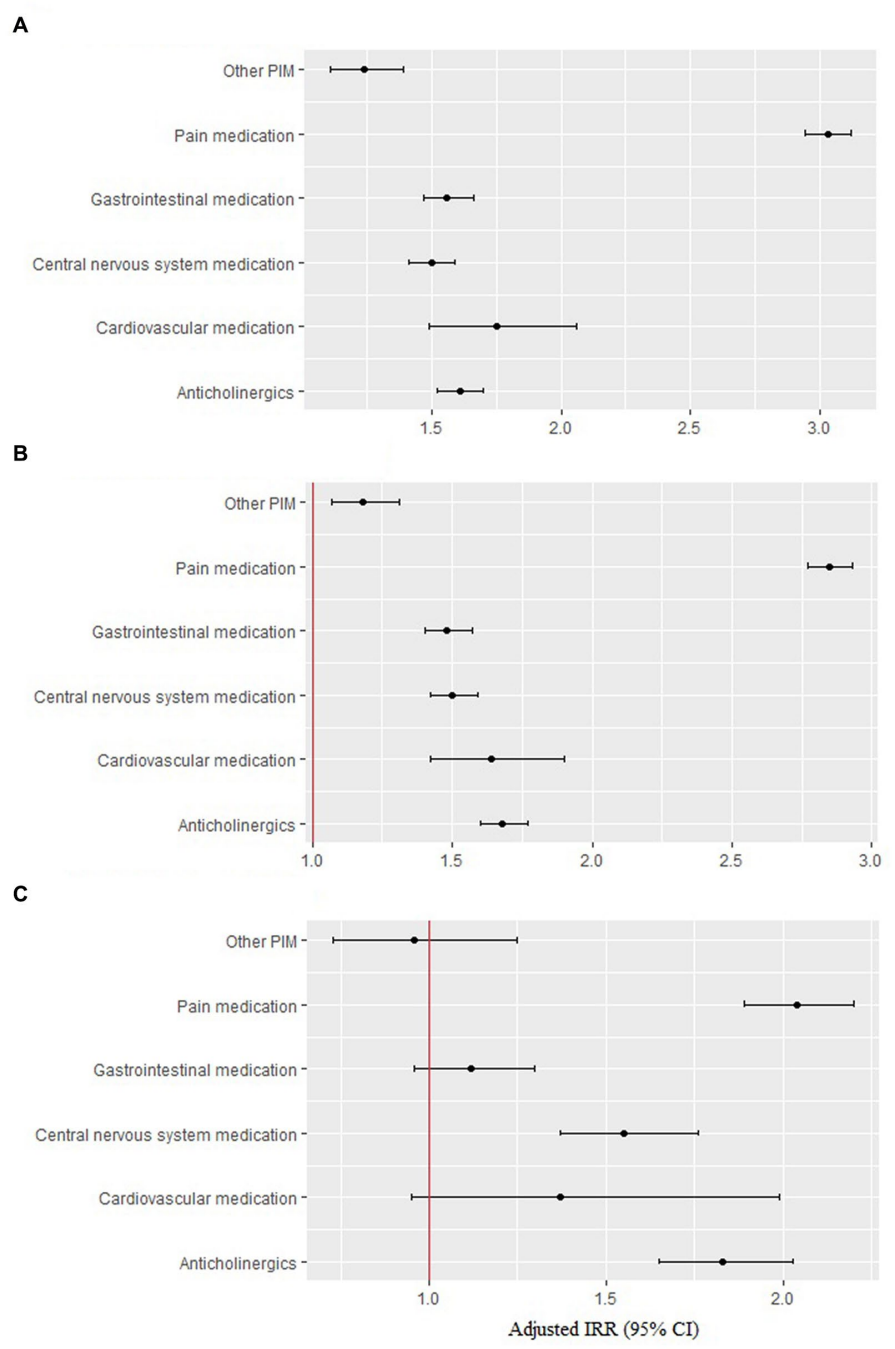
Risk of hospitalization and ED visits by each PIM category. **(A)** All events (*n* = 40,479). **(B)** Hospitalization (*n* = 35,527). **(C)** ED visits (*n* = 11,771).

Among the PIM categories, pain medication was used the most, by 89.5% of individuals, followed by anticholinergics (82.72%) and gastrointestinal (GI) medications (54.13%) (Table 5). When we distinguished the PIM exposure by PIM drug categories and compared their effects with controls, all PIM categories presented a significantly increased risk of hospitalization and ED visits, with risks ranging from 1.18 (other PIM) and 1.68 (anticholinergics) to 2.19 (pain medication), even after adjusting for all time-variant covariates in the Poisson regression model (Figure 3).

**Table 5:** Information of exposures and risk of hospitalization and ED visits by PIM category.

PIM exposures by PIM category	
Anticholinergics (*N*, %)	36,349 (82.72)
Cardiovascular (*N*, %)	2,346 (5.34)
Central nervous system (*N*, %)	17,625 (40.11)
Gastrointestinal (*N*, %)	23,785 (54.13)
Pain medications (*N*, %)	39,322 (89.49)
Other PIM (*N*, %)	6,583 (13.98)

*Other PIM categories included anti-infectives, antithrombotics, genitourinary, and endocrine medications.

### 3.4. Subgroup analysis stratified by the number of co-medication

We conducted subgroup analysis to examine the PIM effects stratified by the numbers of co-medication. All results remained similarly significant in hospitalization and ED visits but the PIM effects seemed to decrease gradually depending on the increasing number of co-medications demonstrating that PIM and polypharmacy collectively affected the outcome events (Table 6).

**Table 6:** Subgroup analysis by number of comedication.

Periods	Adjusted IRR by number of comedication group			
	0 (*n* = 12,622)	1–4 (*n* = 19,136)	5–9 (*n* = 10,222)	10+ (*n* = 1,962)
All events				
Non-exposure	1	1	1	1
Exposure	2.24 (2.19, 2.3)	2.18 (2.14, 2.23)	1.85 (1.8, 1.9)	1.62 (1.54, 1.71)
Post-exposure	1.44 (1.39, 1.49)	1.42 (1.38, 1.46)	1.47 (1.41, 1.52)	1.35 (1.24, 1.46)
Hospitalizations				
Non-exposure	1	1	1	1
Exposure	2.26 (2.19, 2.33)	2.19 (2.14, 2.24)	1.87 (1.82, 1.93)	1.57 (1.48, 1.66)
Post-exposure	1.59 (1.53, 1.66)	1.53 (1.48, 1.58)	1.6 (1.54, 1.67)	1.39 (1.28, 1.52)
ED visits				
Non-exposure	1	1	1	1
Exposure	1.86 (1.76, 1.97)	1.84 (1.75, 1.93)	1.55 (1.46, 1.64)	1.55 (1.37, 1.76)
Post-exposure	0.91 (0.83, 0.99)	0.97 (0.9, 1.04)	0.94 (0.85, 1.03)	1.01 (0.82, 1.24)

### 3.5. Sensitivity analysis

We also performed a sensitivity analysis to confirm the robustness of the results of this study, using only the first exposure episode and its outcome. We found a similar trend in all risk estimations for the outcome events. After adjusting for time-variant covariates, the model remained significant in all evaluations, and the magnitude of the risk was similar to the results including all recurrent exposures, as presented in Table 7.

**Table 7:** Sensitivity analysis using only first exposure episode.

Periods	All events (*n* = 43,942)		Hospitalizations (*n* = 38,701)		ED visits (*n* = 12,695)	
	Crude IRR	Adj. IRR	Crude IRR	Adj. IRR	Crude IRR	Adj. IRR
3-levels						
Non-exposure	1	1	1	1	1	1
Exposure	2.19 (2.14, 2.25)	1.97 (1.88, 2.06)	2.22 (2.15, 2.28)	2 (1.92, 2.09)	1.68 (1.58, 1.78)	1.58 (1.39, 1.81)
Post-exposure	1.54 (1.48, 1.60)	1.49 (1.41, 1.57)	1.67 (1.6, 1.75)	1.66 (1.58, 1.75)	0.98 (0.88, 1.08)	0.89 (0.75, 1.06)
7 levels						
Non-exposure	1	1	1	1	1	1
Exposure1	4.12 (3.95, 4.30)	4.07 (3.86, 4.29)	4.31 (4.11, 4.51)	4.3 (4.09, 4.53)	3.12 (2.82, 3.45)	3.01 (2.57, 5.53)
Exposure2	2.6 (2.41, 2.79)	2.43 (2.23, 2.65)	2.62 (2.41, 2.84)	2.49 (2.28, 2.72)	2.2 (1.86, 2.6)	2.1 (1.66, 2.68)
Exposure3	2.3 (2.14, 2.47)	2.08 (1.92, 2.27)	2.38 (2.2, 2.57)	2.18 (2, 2.38)	1.68 (1.41, 2.01)	1.58 (1.26, 1.98)
Exposure4	1.62 (1.56, 1.67)	1.37 (1.29, 1.44)	1.61 (1.55, 1.68)	1.37 (1.3, 1.44)	1.24 (1.14, 1.35)	1.13 (0.98, 1.31)
Post1	1.9 (1.8, 2)	1.9 (1.78, 2.02)	2.09 (1.98, 2.21)	2.12 (2, 2.25)	1.11 (0.97, 1.27)	1.06 (0.87, 1.28)
Post2	1.18 (1.11, 1.26)	1.17 (1.08, 1.26)	1.25 (1.17, 1.34)	1.27 (1.17, 1.36)	0.84 (0.72, 0.99)	0.8 (0.64, 0.98)

*Exposure1(day0–day14), exposure2 (day15–day28), exposure3 (day29–day56), and exposure4 (day57–day_end).

## 4. Discussion

In this study, we found that compared to the non-exposure period, the risk of hospitalization and ED visits increased by 1.99 and 1.41 times during the exposure and post-exposure periods using the SCCS model after adjusting for potential time-variant covariates over an average of 1,352 follow-up d (3.70 y). These results were similar to, albeit with a bit of reduced risk, that of a previous Korean study that reported that the risk of hospitalization (odds ratio 2.25, 95% CI 2.09–2.44) and ED visits (odds ratio 1.59, 95% CI 1.50–1.67) was higher in older adults who took at least one PIM than in controls (24). The slight difference may be due to variations in the study design and subject characteristics, and older adults included in this study may have been comparatively healthier at cohort enrollment because they were required to satisfy the 6 month non-PIM user criteria (see Figure 1).

The US retiree health care data presented similar risk with this study that taking one or more of the PIM by Beers or NCQA lists were 1.8 to 1.9 times more likely to have a hospital admission after adjusting for age, sex, number of prescriptions overall, and comorbid disease severity (25). The recent systemic review and meta-analysis also reported that PIM was associated with increased odds of adverse drug event-related hospital admissions (adjusted OR 1.91, 95% CI 1.21–3.01) (26).

In total, older adults in this study had 261 d (0.72 per y) of PIM exposure, and had multiple exposure periods, mean number of exposure periods was 5.67 during the whole study period meaning PIM prescriptions were predominant, recurrent, and consistent. The risk of all outcomes decreased gradually when the exposure period was divided by PIM prescription days. Exposure 1 (day 1–14) had the highest risk of outcome events, presenting 3.29 times increased risk, which reduced gradually to 1.17 times in post exposure period 2. Based on these findings, older adults who tolerated longer periods of PIM prescriptions might have a reduced risk of hospitalization or ED visits attributable to PIM. Residual effects still existed, given that the post-exposure period also showed an increased risk. Therefore, follow-up monitoring is still needed after discontinuation of PIM in older adults, and close monitoring and introduction of patient education regarding PIM-related adverse events should be practiced in the early period of PIM prescriptions in older adults.

The highest risk was caused by pain medication, which was prescribed to 89.5% of older adults, followed by anticholinergics (82.72%) and then GI tract medications (54.13%).

Pain medications included nonsteroidal anti-inflammatory drugs (NSAID), muscle relaxants, and narcotic analgesics (pethidine and pentazocine). Clinical guidelines from medical societies, including the American Geriatric Society (AGS), recommend using NSAIDs with caution and limiting their use to the lowest effective dose and shortest duration. When NSAIDs are used, common gastrointestinal, renal, and cardiovascular side effects should be routinely monitored (27–29). Chronic use of all NSAIDs, including high dose aspirin, should be avoided because of the risk of gastrointestinal bleeding (30), which was four fold in the older adults (31). High cardiovascular risk (32) and renal side effects of NSAIDs are major concerns in older adults owing to vasoconstriction and reduced renal perfusion via inhibition of prostaglandin and thromboxane synthesis by NSAID. It can eventually induce electrolyte imbalance, edema, high blood pressure, chronic kidney disease, acute interstitial nephritis, and renal papillary necrosis, and reduce the glomerular filtration rate (33). However, NSAID-induced gastroduodenal ulcers can be prevented by the use of GI protective agents, such as misoprostol, H2-receptor antagonists (H2RA), or proton pump inhibitors (PPI) (34). This might explain why a high rate of GI medications were prescribed to older adults in this study.

Gastrointestinal drugs are composed of gastric antispasmodic metoclopramide and proton pump inhibitors (PPI, dexlansoprazole, esomeprzole, etc.). Gastrointestinal antispasmodic drugs are highly anticholinergic, but still play many roles in the treatment of older adults. Proton pump inhibitors were also sometimes prescribed to patients without gastrointestinal hemorrhage or peptic ulcers (4).

Anticholinergic-acting medications are commonly prescribed to approximately one-third of older adults in the primary care population (35). In fact, a study using the Beers criteria found that 39.9% of older adults with dementia on an outpatient basis were prescribed anticholinergic drugs classified as potentially inadequate (36). The side effects of anticholinergic drugs are related to their action on central and/or peripheral cholinergic receptors (37) and vary depending on the anticholinergic drug load and individual vulnerability. Anticholinergics are associated with chronic comorbidities (38), urinary incontinence, arterial hypertension (39), impaired health status, and anxiety and mood disorders (40). In addition, anticholinergics are associated with worse cognitive and functional performance in a dose-response pattern (41). Among patients with mild cognitive impairment (MCI) or dementia treated in memory clinics, 44.7% were taking anticholinergic drugs, and 11.7% received a high anticholinergic load (42). Given that the burden of Alzheimer’s disease and dementia is increasing rapidly worldwide due to aging (43), anticholinergic prescriptions in older adults will require additional safety precautions.

However, for certain clinical syndromes, the benefits of anticholinergics are greater than their risks, and their prescription can be considered adequate for the older adults in some cases, such as psychotropic drugs (35). In nursing homes, the most prevalent anticholinergics were cardiovascular drugs, followed by antipsychotics and antidepressants (44, 45).

Deprescribing is the process of tapering or stopping drugs to minimize polypharmacy and improve patient outcomes. Evidence for the efficacy of deprescribing has emerged from randomized trials and observational studies. The main strategies are drug reconciliation, drug prioritization by benefit-risk assessment, implementation of discontinuation regimens, and patient monitoring plans (46, 47). Based on a previous study, the willingness of older adults with polypharmacy towards deprescribing was not associated with PIM use. These results suggest that patients may be unaware of PIMs. This implies the need to raise patients’ awareness about PIMs through education to implement deprescribing in daily practice (48). Interestingly, in this study, when we stratified the PIM use outcomes by the number of co-medications, the PIM effects on the outcome of interests were attenuated by the increased number of co-medications. This result implies that not only PIM but also polypharmacy collectively results in adverse drug events in older adults which warrants further consideration of deprescribing strategy.

Meanwhile, several randomized clinical trials have demonstrated the efficacy of pharmacist interventions in correcting PIMs (49, 50), long-term discontinuation of PIMs (51), and reduction in the number of medications prescribed (52). As the prevalence and type of PIMs vary by country and healthcare setting (53), contextualized measures based on these variations should be developed in each country.

We implemented the SCCS method, which provided an alternative epidemiological study design to investigate the association between transient exposure and an outcome event. The method allows only cases to be included in the study and has the advantages that no separate controls are required and any fixed confounder is automatically controlled (21). We observed an increased risk in hospitalizations and ED visits in older adults due to PIM use and risk differentiation in PIM categories and PIM exposure period stratification.

Nevertheless, the study has several limitations. First, since we selected patients who did not have a PIM prescription for 6 months before cohort enrollment to account for new PIM users, the enrolled individuals were comparatively healthy with low CCI scores and low rates of chronic complex conditions. Thus, the results of this study may be an underestimation of real-world effects. Second, due to the base of SCCS only comprising cases can have limitations such as not portraying the comparison from control subjects. Third, we sought to represent the general overview of PIM outcomes in older adults using PIM categories in the Beers criteria 2019 (Supplementary Table S1), however, the other criteria in others in Beers 2019 should be studied further as well to delineate PIM use outcomes in older adults in detail. Fourth, we used the psychiatric drug prescription record to capture psychiatric diseases due to the limitation of insurance claim data which does not provide highly confidential psychiatric disease ICD codes in Korea, which might overestimate the numbers of comorbid psychiatric diseases in our datasets based on potential off-label use of psychiatric drugs. We tried to find the off-label psychiatric drug use trend in older adults in Korea, however only articles on off-label antidepressant use among adolescents and pediatric patients were found (54–56). They reported prevalent off-label antidepressant use in these populations, and we can assume off-label psychiatric drug use might be prevalent in older adults as well as reported by previous studies from other countries (57, 58). However, the adjusted model overall presented similar results to the unadjusted model in this study, the covariate overestimation might not count toward altering the results. Finally, due to the nature of recurrent PIM use in older Korean adults, the essential SCCS requirement that exposure should not affect subsequent exposures was not fully guaranteed. However, by performing sensitivity analyses by only including the first PIM exposure, we portrayed results similar to those of the analysis with recurrent and consistent PIM exposures.

## 5. Conclusion

Hospitalization and ED visits were greatly increased following PIM use in older adults. Thus, close monitoring of PIM use in older adults and implementation of deprescribing strategies for PIM use in the future are strongly recommended.

## Data availability statement

Publicly available datasets were analyzed in this study. This data can be found here: https://nhiss.nhis.or.kr/bd/ab/bdaba022Oeng.do National Health Insurance Service-Elderly Cohort Database (NHIS-ECDB).

## Author contributions

JL: conceptualization, data analysis, and manuscript revision. SohJ: conceptualization, manuscript draft, and revision. SuhJ: conceptualization and manuscript revision. SunJ: funding acquisition, conceptualization, and manuscript revision. All authors contributed to the article and approved the submitted version.

## Funding

This research was funded by the Mid-Career Researcher Program of the National Research Foundation of Korea (2020R1A2C1008563). The funders had no role in the study design, data collection, analysis, decision to publish, or manuscript preparation.

## Conflict of interest

The authors declare that the research was conducted in the absence of any commercial or financial relationships that could be construed as a potential conflict of interest.

## Publisher’s note

All claims expressed in this article are solely those of the authors and do not necessarily represent those of their affiliated organizations, or those of the publisher, the editors and the reviewers. Any product that may be evaluated in this article, or claim that may be made by its manufacturer, is not guaranteed or endorsed by the publisher.
